# An evolutionarily biased distribution of miRNA sites toward regulatory genes with high promoter-driven intrinsic transcriptional noise

**DOI:** 10.1186/1471-2148-14-74

**Published:** 2014-04-04

**Authors:** Hossein Zare, Arkady Khodursky, Vittorio Sartorelli

**Affiliations:** 1 Laboratory of Muscle Stem Cells and Gene Regulation, National Institute of Arthritis, Musculoskeletal and Skin Diseases, National Institutes of Health, 50 South Drive, Bethesda, MD 20892, USA; 2 Department of Biochemistry, Molecular Biology & Biophysics, Biotechnology Institute, University of Minnesota, 140 Gortner Labs, 1479 Gortner Avenue, St. Paul, MN 55108, USA

**Keywords:** microRNAs, Transcriptional noise, Nucleosome occupancy, Evolution

## Abstract

**Background:**

miRNAs are a major class of regulators of gene expression in metazoans. By targeting cognate mRNAs, miRNAs are involved in regulating most, if not all, biological processes in different cell and tissue types. To better understand how this regulatory potential is allocated among different target gene sets, we carried out a detailed and systematic analysis of miRNA target sites distribution in the mouse genome.

**Results:**

We used predicted conserved and non-conserved sites for 779 miRNAs in 3′ UTR of 18440 genes downloaded from TargetScan website. Our analysis reveals that 3′ UTRs of genes encoding regulatory proteins harbor significantly greater number of miRNA sites than those of non-regulatory, housekeeping and structural, genes. Analysis of miRNA sites for orthologous 3′UTR’s in 10 other species indicates that the regulatory genes were maintaining or accruing miRNA sites while non-regulatory genes gradually shed them in the course of evolution. Furthermore, we observed that 3′ UTR of genes with higher gene expression variability driven by their promoter sequence content are targeted by many more distinct miRNAs compared to genes with low transcriptional noise.

**Conclusions:**

Based on our results we envision a model, which we dubbed “selective inclusion”, whereby non-regulatory genes with low transcription noise and stable expression profile lost their sites, while regulatory genes which endure higher transcription noise retained and gained new sites. This adaptation is consistent with the requirements that regulatory genes need to be tightly controlled in order to have precise and optimum protein level to properly function.

## Background

Robustness, the ability of a system to sustain internal and external perturbations, is deemed essential for the integrity of biological systems, including those associated with organismal development and responses to environmental stimuli. At the cellular level, gene expression is one of the primary factors defining molecular and cellular outcomes such as the acquisition of specific cell fates. Because of its stochastic nature and high sensitivity to external perturbations, gene expression is highly dynamic and this feature has been considered one of the main factors affecting biological robustness [1-3]. To overcome transcriptional noise, several regulatory mechanisms are in place to control gene expression dynamics and maintain optimal protein levels.

Post-transcriptional regulation of messenger RNAs (mRNA) by micro-RNAs (miRNA) has been identified as an important mechanism conferring biological robustness and dampening gene expression noise [4-6]. miRNAs are a class of small non-coding RNAs that can repress gene expression by complementary base paring of their seed region to their target sites located at the 3′ UTR of mRNAs [7,8]. Most mammalian mRNAs are targeted by miRNAs and post-transcriptionally regulated through the presence of either conserved or non-conserved miRNA sites in their 3′ UTR [9]. Thus, numerous biological processes and functions are influenced by miRNAs, as evidenced by their involvement in regulating normal physiological as well as pathological conditions [10-12]. While as many as ~50% of mammalian mRNAs appear to be under selective pressure to maintain pairing to miRNAs through conserved sites [9], there are many more functional but non-conserved miRNA sites that can also potentially affect multiple biological processes [13-16]. A significant fraction of non-conserved miRNA sites can be explained by a selective avoidance model, which states that non-conserved sites are often found in 3′ UTR of genes, which are expressed in tissues where the cognate miRNA is absent [13]. However, the model does not explain the many instances of miRNA sites [17], which complicates the interpretation of the landscape of miRNA and mRNA interactions.

Maintaining an optimal protein concentration is essential in most biological processes, and that deviation from an appropriate protein level can be detrimental to the cell. miRNAs have emerged as primary regulatory elements to fine tune gene expression levels and maintain the protein production at their optimal levels by filtering and buffering transcription noise and unintended fluctuations in gene expression [4,5,18-21]. However, the depth and breadth of this functionality and whether there exists an evolutionary miRNA target recognition bias toward particular classes of genes, remains to be systematically investigated. In order to address this issue, we deployed a bottom-up approach to study the landscape of all predicted conserved and non-conserved miRNA-mRNA interaction sites in the mouse genome. To that end, we used predicted conserved and non-conserved sites for 779 miRNAs in 3′ UTR of 18440 genes generated by TargetScan tool (http://www.targetscan.org). Additionally, we incorporated miRNAs sites of orthologous 3′ UTR for 10 other species along with 3‘ UTR and gene conservation score for comparative analysis. Furthermore, a high throughput gene expression meta-data set and promoter sequence features of mouse genes were utilized to deduce gene expression variability and transcription noise.

Here, we report that the 3′ UTRs of genes whose products are involved in regulatory processes such as transcription factors (TFs), contain on average more sites for miRNAs compared to those in housekeeping and non-regulatory genes. Furthermore, the likelihood of genes with higher expression variability and inherent expression noise to be targeted by miRNAs is significantly higher than that of genes with stable expression level. Interestingly, the extent of miRNAs targeting 3′ UTR of a gene is linearly correlated with the extent of gene expression noise driven by its promoter DNA sequence content. We propose an evolutionary conjecture that may explain the presence of widespread interaction between miRNAs and mRNAs in distant species. Specifically, the results of our analyses reveal that the 3′ UTR of genes that are more resistant to transcriptional fluctuations have in aggregate lost miRNA sites whereas, likely as a result of selective pressure, the 3′ UTR of regulatory genes and genes with higher expression noise have on average gained additional miRNA sites, potentially stabilizing their inherent fluctuation rate.

## Results

### The 3′ UTR of regulatory genes are enriched for miRNAs recognition sites

**Figure 1:**
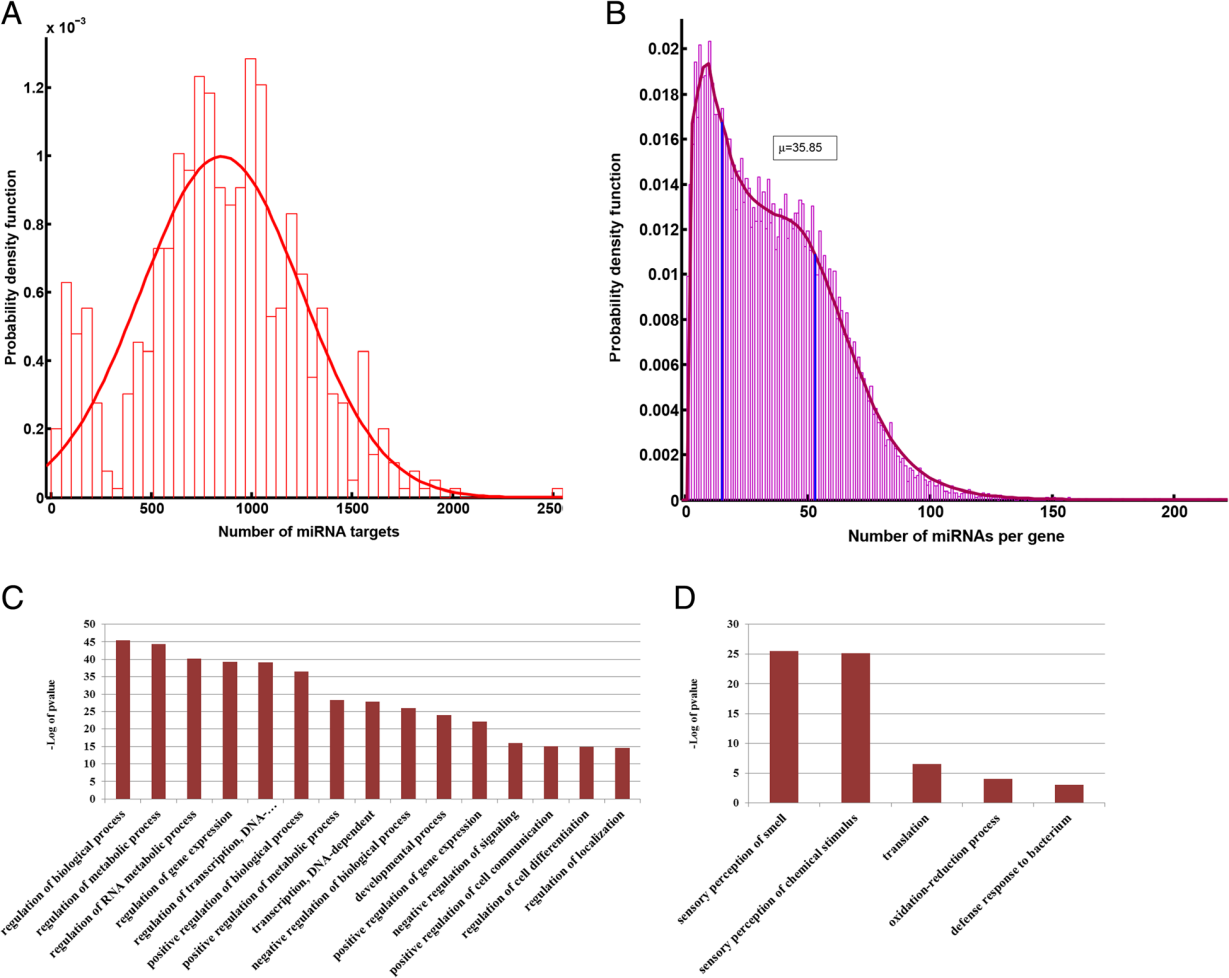
**Genes involved in regulatory processes are disproportionally targeted by miRNAs. (A)** Distribution of miRNAs targets. **(B)** Distribution of number of distinct miRNA targeting 3′ UTR of genes. The two vertical lines (in blue) indicate 25 (left line) and 75 (right line) percentiles values. **(C)** A list of the over-represented GO biological process terms in the set of genes with more than 53 predicted miRNA sites in their 3′ UTR ( 4th quartile set). **(D)** A list of the over-represented GO biological process terms in the set of genes with less than 15 predicted miRNA sites in their 3′ UTR ( 1st quartile set).

For a genome wide analysis of miRNA targets, we employed the TargetScan Mouse data sets of ‘Conserved site context + score’ and ‘Nonconserved site context + score’ (Methods). The distributions of the number of miRNA targets and number of distinct miRNAs sites per genes are illustrated in Figure 1-A and B respectively. A single miRNA can target many genes, and conversely a single gene can be targeted by several distinct miRNAs [9]. However, for many genes, the total number of miRNAs targeting their 3’ UTR exceeds the expected value, prompting an inquiry into the significance of this phenomenon. The absolute number of binding sites for each gene is highly correlated with the number of miRNAs targeting that gene, and therefore, as it is detailed in the method section, in our analysis we counted multiple sites of the same miRNA in 3′ UTR of the gene as one binding sites (Additional file 1: Figure S1). We started our analysis by defining two groups of genes. The first group includes genes with low number of miRNAs sites (less than 15- 1st quartile), while the second group consists of genes with high number of miRNAs sites (more than 53- 4th quartile). Comparative functional analysis on these two groups returned GO terms significantly enriched in the 4th quartile gene list (Figure 1-C). These include “regulation” of biological process, of transcription and gene expression, of metabolic process, “regulation” of localization and etc. Additionally, the 4th quartile gene list was enriched for GO-cellular components such as; intracellular part, neuron projection and synapse, cell junction, etc. (Additional file 1: Figure S2-A). Moreover, GO-molecular functions such as sequence specific DNA binding, protein binding, chromatin binding were among the highly significant categories (Additional file 1: Figure S2-B).

Examination of the 1st quartile gene list revealed that, unlike the 4th quartile, these genes were not involved in regulatory processes, and specifically the list was enriched for terms such as sensory perception of smell and stimuli, extracellular regions, ribosome, olfactory receptor activity, among others (Figure 1-D, and Additional file 1: Figure S2-C,D). Complete GO analysis results and the list of predicted miRNAs for genes are provided in supplementary table (Additional file 2).

Further analysis of all enriched categories and genes belonging to them confirmed that the 4th quartile group comprises mainly regulatory genes (i.e., genes with regulatory functions are likely to be targeted by many distinct miRNAs.) To test this hypothesis, we evaluated whether TFs, well-known elements of gene regulatory networks, are preferentially enriched in the 4th quartile set. Indeed, we found that TFs are enriched in the upper quartile group (4th quartile) (Hypergeometric, p-value = 5.1e-12), and depleted in the lower quartile group (1st quartile) (Hypergeometric, p-value = 3.68e-20). Moreover, 3′ UTRs of TF mRNAs have, on average, more miRNA sites compared to non-TF genes (Wilcoxon rank sum test, p-value = 5.6e-26). Additionally, the 3′ UTRs of tissue specific TFs contain sites for a larger number of distinct miRNAs than that of non-TF tissue specific genes (Additional file 1: Figure S3-A). Similarly, tissue specific genes of highly complex organs, like the brain, are targeted by more distinct miRNAs (Additional file 1: Figure S3-B).

To verify that the above results, derived from *de novo* predicted miRNA sites, accurately reflect the *in vivo* biological regulation by miRNAs, we utilized CLIP-Seq data, the *in vivo* data uncovering mRNA-miRNA interactions. To that end, we used a publicly available Argonaut HITS-CLIP data set (GSE41285) [22]. Total of 4165 genes were identified as being targeted by miRNAs, out of which 937 genes were noted to have more than 5 distinct miRNAs sites. Majority of these genes (70%) fall in the 4th quartile group. Furthermore, GO analysis of these 937 genes revealed that the list is enriched for categories such as regulation of transcription, DNA binding, transcription factor activity, regulation of RNA metabolic process, etc. (Additional file 3). Although this data set is limited and represents only an instance point in a multi-dimensional space of possible regulatory interactions of miRNAs, it indicates that regulatory genes which are predicted to be targeted by many distinct miRNAs are also more likely to be targeted by miRNAs *in vivo*. Altogether, these observations are consistent with a role of miRNA in fine-tuning gene expression, and indicate that genes involved in regulatory processes have an increased probability to harbor miRNA binding sites in their 3′UTRs, perhaps to insure a steady-state level of their corresponding mRNAs.

### Genes with higher expression variability are preferentially targeted by several distinct miRNAs

The observation that regulatory genes and TFs more frequently undergo post-transcriptional regulation than other genes prompted us to postulate that these genes may have higher expression variability across different conditions. If this assumption is correct, it should be possible to capture higher expression variation for this group of genes by employing a mega data set of expression profiles across many experimental conditions. We tested this possibility by parsing the public ArrayExpress database for mouse transcriptional data (http://www.ebi.ac.uk/arrayexpress/). We used the E-MTAB-28 dataset, an integrated, high quality mouse expression data across 886 samples (http://www.ebi.ac.uk/arrayexpress/experiments/E-MTAB-28/) [23].

To assess the variability of transcription across samples, we calculated a measure of expression variability for each gene as the level of variability in relation to the average expression profile, or what is commonly known as the coefficient of variation. Coefficient of variation is defined as the ratio of the standard deviation to the mean,

$$\vartheta =\frac{\sigma }{\mu }$$

**Figure 2:**
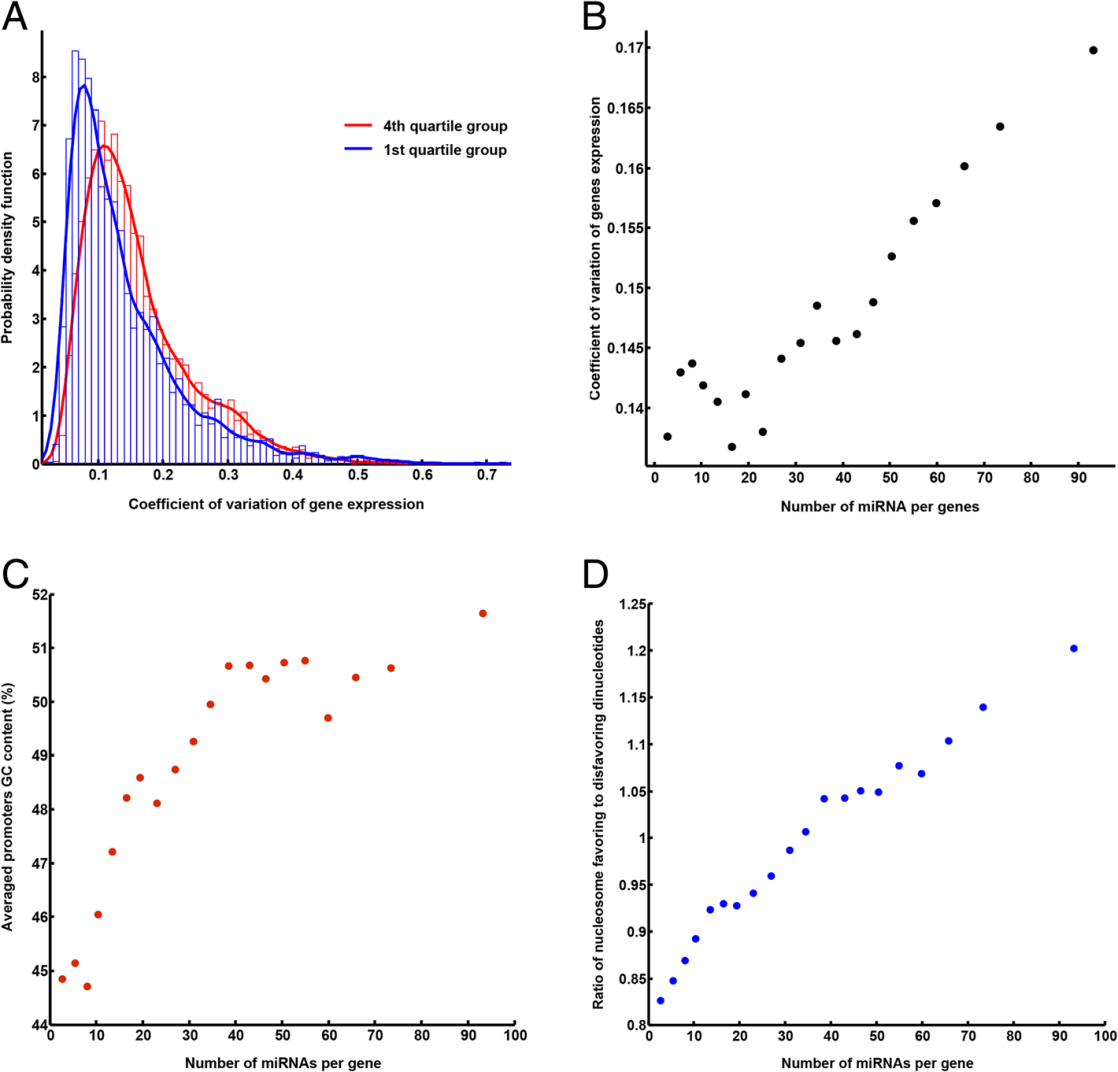
**Genes with higher expression variability are targeted by more miRNAs. (A)** Distribution of the coefficient of variation values for gene expression for 1st and 4th quartile groups. The coefficient of variation for each gene was calculated from meta-data set of gene expression profile across 886 arrays. **(B)** A scatter plot of the average gene expression variability versus the average number of distinct miRNA sites for vigintiles. Vigintiles were created by ranking genes based on the number of miRNA sites in their 3′ UTR. **(C)** A scatter plot of the average promoter GC content versus the average number of distinct miRNA sites for vigintiles. **(D)** A scatter plot of the average ratio of nucleosome favoring to disfavoring di-nucleotides versus the average number of distinct miRNA sites for vigintiles.

Next, we compared the distribution of this measure for two groups of genes, previously referred to as the 1st and 4th quartile groups. Interestingly, we observed statistically significant difference in distributions of the variability measure between two groups (p-value = 1.68e-54, Kolmogorov-Smirnov test) (Figure 2-A). Moreover, a significant correlation was observed between the extent of gene expression variability and the number of distinct miRNA targeting the 3′ UTR region (Figure 2-B).

It has been shown that promoter sequence composition, which can affect the number and affinity of TF binding sites as well as nucleosome occupancy, is greatly responsible for transcriptional noise [24-26]. Along the same line, it has been demonstrated that lowering nucleosome occupancy by adding nucleosome-disfavoring sequences to the promoter reduces expression variability and noise [26-28]. Furthermore, the GC content of a sequence is a prominent factor defining nucleosome occupancy [29]. Therefore, the GC content of promoters can be employed as a good predictor of gene expression noise, so that the higher promoter’s GC content, the higher the expression variability. Notably, we found that genes with higher GC content in their promoter sequences have higher number of miRNA sites in their 3′ UTR (Figure 2-C and Additional file 1: Figure S4-A). Similar results were observed when considering the ratio of nucleosome favoring (GC, CG, GG) and disfavoring (AA, CA, AC) di-nucleotide usage in the promoter sequences of these two groups of genes (Figure 2-D, and Additional file 1: Figure S4-B). Furthermore, as we would expect, similar relationship between transcriptional variability and number of miRNAs targeting 3′ UTR was observed when using miRNAs predicted sites from a different algorithm like miRanda [30] instead of TargetScan (Additional file 1: Figure S5-A-D)

Gene expression noise and variability can be reduced through a negative feedback mechanism [31,32]. If the expression of miRNAs is coherently coupled with that of their target mRNAs encoding regulatory proteins, it guarantees that their interaction with target mRNAs reduces transcriptional noise, thus stabilizing gene expression. Coherent transcriptional regulation of miRNA and target regulatory genes would be expected to require significant co-localization of miRNAs near to or in intronic regions of regulatory genes. Indeed, we found that among 568 annotated miRNAs in UCSC genome browser website (mm9 assembly), 417 miRNAs are co-localized within -/+20 kb of 480 genes. When we subjected the identified genes to GO analysis, genes involved in regulation of biological process and gene expression were significantly and prominently over-represented (Additional file 1: Figure S3-C). Overall these results suggest that regulatory genes are often subjected to greater transcriptional variability, which imposes the requirement for a correction mechanism. Fine-tuning gene expression by means of miRNAs can fulfill this requirement, which in turn teleologically warrants the selective inclusion of a greater number of miRNA sites in the 3′ UTR of regulatory genes.

### The 3′ UTR of regulatory genes are most conserved

**Figure 3:**
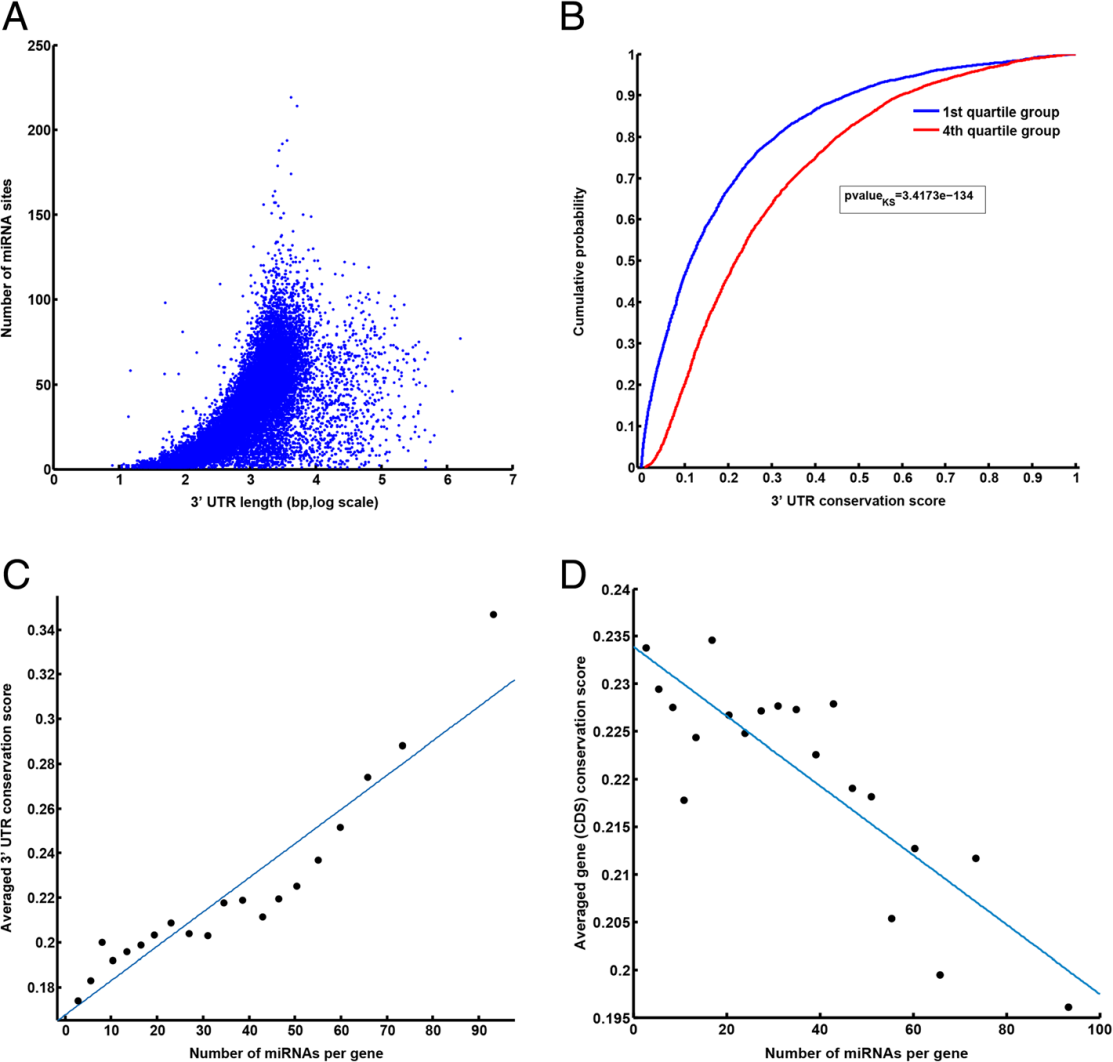
**3**′ **UTR of regulatory genes harboring multiple distinct miRNA sites are more conserved compared to other genes. (A)** Relation between the 3′ UTR length and number of miRNA sites; each dot represent a gene. **(B)** Distribution of 3′ UTR sequence conservation scores for 1st and 4th quartile group. **(C)** A scatter plot of the average 3′ UTR conservation score versus the average number of distinct miRNA sites. **(D)** A scatter plot of the average of conservation score of gene coding region versus the average number of distinct miRNA sites.

To further understand the apparent miRNA recognition bias toward mRNA of regulatory genes, we first questioned whether a greater number of miRNA sites could be explained based on the length of 3′ UTR regions of regulatory genes. Although we observed, confirming an earlier report [33], a positive correlation between the 3′ UTR length and the number of distinct miRNA sites (Figure 3-A), the length alone could not explain the presence of a larger number of miRNA sites at 3′ UTR of regulatory genes. We found that 3′ UTR regions containing repeat elements such as LTR and SINE are longer than the 3′ UTRs devoid of these elements (Additional file 1: Figure S6-A). Also, 3′ UTRs which contain repeat elements have the median number of distinct miRNA sites equals to 45 compared to the median of 24 sites for 3′UTRs without repeat elements (Additional file 1: Figure S6-B). Consequently, higher fraction of genes in the 4th quartile group contains LTR/SINE elements compared to the 1st quartile groups (60% vs. 17%). Nonetheless, within the 4th quartile group, the median number of distinct miRNA sites was not higher for LTR/SINE containing 3′UTRs than that for 3′ UTRs devoid of LTR/SINE elements (66 sites vs. 66 sites). Therefore, within the 4th quartile group we could not find statistically significant association between the presence of the repeat elements or length and the number of miRNA sites (p-value = 0.15, Wilcoxon rank sum test). For some of these 3′UTRs, the repeat elements may explain the presence of other regulatory elements such as binding sites for DNA or RNA binding proteins [34,35]. A useful approach to interrogate element functionality relies on sequence conservation [36-38]. Therefore, we evaluated the relationship between 3′UTRs sequence conservation score and the number of miRNA sites. The sequence conservation scores, calculated by multiple alignment of 30 vertebrates genome sequences using *PhastCons* method [39], were downloaded from UCSC genome browser website. The results of this analysis revealed a shift to the right in the distribution of the 3′UTR conservation score for 4th quartile genes compared to 1st quartile group, which suggests that the 3′ UTRs of genes in upper quartile group are more conserved than those of the lower quartile group (Figure 3-B). This positive correlation was even more evident when plotting the average 3′ UTR conservation score versus the average number of distinct miRNA sites for each of the 20 vigintiles (Figure 3-C). Unexpectedly, the correlation between the number of miRNA sites and the conservation score of regions encompassing the coding region of genes was negative (Figure 3-D). Together these results suggest that, even though the coding regions of regulatory genes are less conserved when compared to housekeeping and non-regulatory genes, their 3′ UTR regions are better conserved and encompass more miRNA sites.

### Interaction between miRNAs and mRNAs were widespread in distant species

In the preceding sections we have established the case for a significant over-representation of miRNA target sites in the 3′ UTR of regulatory genes. However, how such selective mechanism could have evolved remains to be explained. As part of an effort to address this question, we seized on our observation that 3′ UTR regions of non-regulatory genes show lower conservation than their coding regions (Figure 3). Such relative diversity of the 3′ UTRs can be due to a higher rate of accumulated mutations, which in turn may result in scrambling of miRNA target sites. To qualitatively evaluate this possibility, we hypothesized that the bias in the distribution of miRNA sites is a result of the evolutionary process, where originally unbiased distribution of miRNA sites among all genes independent of their function resulted in a relative loss of the sites among non-regulatory genes and conservation or even gain of the sites among regulatory genes.

**Figure 4:**
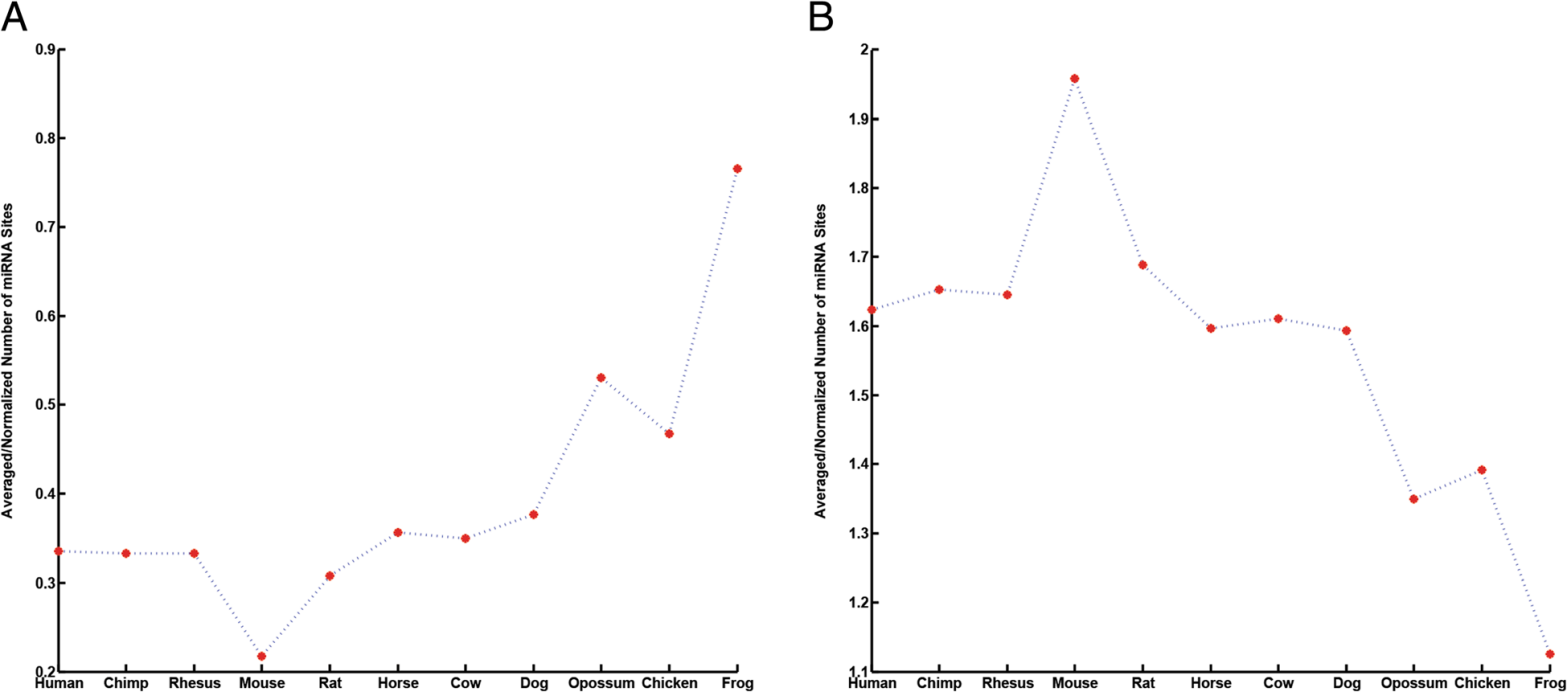
**miRNA target recognition is evolutionarily biased in favor of regulatory genes. (A)** Species-specific loss of miRNA sites for non-regulatory genes (1st quartile). **(B)** Species-specific gain, or retention, of miRNA sites for regulatory genes (4th quartile). Species were ordered based on their evolutionary distance to mouse genome.

To evaluate this conjecture, we investigated whether the 1st quartile genes are targeted by more miRNAs in distant species. To do this we took advantage of predicted miRNAs sites in orthologous 3′ UTR for other species in the same TargetScan data set. In addition to the mouse genome, a reference genome in this study, these data sets include predicted sites for human, chimpanzee, rat, cow, horse, dog, chicken, opossum and frog, among others. Similar to that of mouse, we obtained binary matrices of mRNA-miRNA interactions for orthologous 3′ UTR in other species. In each species, we obtained the number of distinct miRNAs targeting each gene and normalized it to the average number of miRNA sites in that species. This normalization is necessary since these species have different number of identified miRNAs and the comparison across species without normalization is unreliable and uninformative. Our assertion was supported by the observation that the average number of miRNA sites for the 1st quartile gene set incrementally increased with evolutionary distance between each species and mouse (Figure 4-A). To determine whether this result was due to the normalization procedure or a random observation, we examined the 4th quartile gene list and found that the relationship between the number of miRNA sites and the evolutionary distance is opposite to that observed for the 1st quartile group (Figure 4-B). Both results convincingly support our “selective inclusion” model according to which non-regulatory genes lost and regulatory genes gained miRNA sites during evolution.

## Discussions

The discovery of microRNAs has unveiled a new mode of gene regulation for these relatively small molecules with important roles in many cellular processes. Large numbers of miRNAs have been identified across a variety of species, and most are known to interact with mRNAs to degrade, stabilize, or inhibit their translation [4]. It has been shown that miRNAs, by fine-tuning gene expression, play a critical role in cell programming, differentiation, proliferation, and cell death [6,10,12,40]. miRNAs regulate their target mRNAs in a sequence specific manner by complementary pairing of miRNA seed sequence mainly to the 3′ UTR of target transcripts. Since the seed sequence consists of only 7–8 base pairs, it provides the opportunity for a given miRNA to interact with many transcripts. On the other hand, the sequence content of the 3′ UTR of a single transcript offers a platform for interaction with a number of miRNAs. To better understand the nature of distribution of miRNA target sites, we carried out genome-wide and comparative analysis of miRNA targets. We found that transcription factors and genes involved in regulatory processes are preferentially targeted by many distinct miRNAs. The analysis of GO terms associated with genes harboring a large number of miRNA sites revealed that these genes are mainly involved in regulation of biological and cellular processes, i.e., regulatory genes. In contrast, the set of genes with relatively small number of miRNA sites were depleted of regulatory genes and primarily comprised of genes encoding structural cellular and extracellular components. Additionally, transcription factors, a bone fide class of regulatory proteins, are targeted by higher than expected number of miRNAs. This result is consistent with the previous observation that functionally important genes are tightly regulated by miRNAs [33].

When comparing gene expression variability, we found that genes with higher variability and expression noise, which are also enriched for regulatory genes, have a higher chance of being targeted by miRNAs. On the contrary, transcripts of housekeeping genes characterized by stable levels do not require being under constant fine tuning, as indicated by a very low number of distinct miRNA sites in their 3′ UTR. Additionally, we observed that genes with higher transcriptional noise driven by promoter sequence content are targeted by more miRNAs compared to transcript with small noise level, suggesting the existence of coupled or coordinated mechanism for transcriptional and post transcriptional regulation.

These observations complement the notion and further suggest the presence of a feedback loop between miRNAs and regulatory genes such as TFs [41-44]. Transcriptional noise or increased transcription of a regulatory gene may result in increased expression of miRNAs targeting its 3′UTR. By virtue of regulatory genes 3′ UTR encompassing many miRNA sites, it is highly likely that one of these affected miRNAs interacts with the regulatory gene′s mRNA to dampen its expression. In agreement with the existence of a feedback mechanism, we found that significant numbers of annotated miRNAs are located nearby or inside regulatory genes.

To understand how evolution could have affected the interactome landscape of miRNAs and their targets, we analyzed the sequence content of miRNAs targets’ in 3′ UTR and the whole gene regions. In addition, we carried out comparative analysis of miRNAs targets across species for orthologous 3′ UTR regions. We found that the relative degree of conservation of whole gene (or CDS region) and 3′ UTR regions is inversed for two groups of genes with high and low number of miRNA sites. Specifically, genes with a low number of miRNA sites are more conserved than their 3′ UTRs alone, whereas genes with a high number of miRNA sites can be characterized by a greater degree of conservation of their 3′UTR regions. Analysis of orthologous 3′UTR’s revealed that the regulatory genes likely have gained and the non-regulatory genes have lost miRNA sites in the course of evolution. For structural and housekeeping genes, which are expected to be stably expressed across all time and spatial domains, this result is consistent with selective avoidance mechanism postulating that genes have evolved to selectively avoid (or exclude) sites for co-expressed miRNAs [13].

## Conclusions

We deployed a bottom-up approach to systematically study the landscape of all predicted conserved and non-conserved miRNA-mRNA interaction sites in the mouse genome. On the basis of our results, one can envision a model according to which the interaction of miRNAs with mRNAs were widespread early on, but during evolution, through a selective pressure, non-regulatory genes with low transcription noise and stable expression profile lost their sites, while regulatory genes which endure higher transcription noise retained and gained new sites. This adaptation is consistent with the requirements that regulatory genes need to be tightly controlled in order to have precise and optimum protein level to properly function.

## Materials and Methods

We used the current version (release 6.2) of TargetScan mouse data set (http://www.targetscan.org/mmu_61/). TargetScan was originally developed by [45] for prediction of miRNA targets in vertebrates and further improved [9,14,46]. This software predicts biological targets of miRNAs by searching for the presence of conserved 8-mer and 7-mer sites that match the seed region of each miRNA and rank them based on the context + scores of the sites [9,46]. Weak microRNA sites were filtered out, and only sites with context + score above 50 and 75 percentile for conserved and non-conserved sites were considered, respectively. The resulting data were converted into binary matrix of gene-miRNA interactions between 18440 genes and 779 miRNAs. An element of matrix for a given gene and a specific miRNA was set to 1 if a site for the miRNA was present in the mRNA’s 3′UTR. Therefore, several occurrences of sites for the same miRNA at defined 3′ UTR were altogether considered as a single site for that specific miRNA. Similarly, a site predicted to interact with two or more distinct miRNAs is counted twice or more. The binary matrices corresponding to interactions of orthologous 3′ UTR and miRNA in other species (human, chimpanzee, rat, cow, horse, dog, chicken, opossum and frog) were generated in a similar manner from the same data sets. Conserved and non-conserved predicted sites for Mouse miRNAs using miRanda algorithm [30] were downloaded from (http://www.microrna.org). Only data sets with good “mirSVR” score [16] were used in our analysis.

Comparative GO analysis of 1st and 4th quartile miRNA target gene sets was carried out using Gorilla web tool (http://cbl-gorilla.cs.technion.ac.il/) [47], by setting 4th quartile gene set as the list of interest and 1st quartile gene set as a background set and vice versa. Tissue specific gene were downloaded from “TiSGeD: a database of tissue specific genes” [48].

Gene expression variability was estimated using promoter DNA sequence features (surrounding +/-1 kb of transcription start sites (TSS)) of all UCSC annotated genes, mm9 assembly. G + C content and occurrence of nucleosome favoring and disfavoring di nucleotides were used as an substitute to nucleosome occupancy to infer intrinsic gene expression noise [26,28,29,49]. The number of occurrences of nucleosome favoring di-nucleotides GC,CG and GG and nucleosome disfavoring di-nucleotides AA, CA and AC in +/-1 kb of TSS were counted, and the ratio of the total count of nucleosome favoring di-nucleotides to the total count of nucleosome disfavoring di-nucleotides was calculated as a measure of nucleosome occupancy. Gene and 3′ UTR conservation score were downloaded from UCSC genome browser websites. We used data from vertebrate conservation (phastCons30way) primary table for the regions of annotated genes overlapped with consensus coding sequence (CCDS) and 3′UTR regions alone. The mean score columns of tables were used for analysis presented in this paper.

## Abbreviations

miRNA: Micro RNA; mRNA: Messenger RNA; TF: Transcription factor; TSS: Transcription start site; GO: Gene ontology; UTR: Untranslated region; CCDS: Consensus coding sequence; UCSC: University of California, Santa Cruz.

## Competing interests

The authors declare no competing financial interests.

## Authors’ contributions

HZ conceived the project, designed the study, carried out the data analysis and wrote the original draft of the manuscript. AK and VS participated in the design of the study and writing the manuscript. All authors read and approved the final manuscript.

## Supplementary Material

Additional file 1 In Pdf format includes Supplementary Figures 1–6. Click here for file

Additional file 2 **In Excel format includes a table of genes with their targeting miRNAs and tables of full GO categories for 1st and 4th quartiles groups of genes defined based on the number of targeting miRNAs.** Click here for file

Additional file 3 In Excel format includes top miRNAs targets from Argonaut HITS-CLIP data set (GSE41285) and their GO analysis. Click here for file
