# Supplementary material for: An evolutionarily biased distribution of miRNA sites toward regulatory genes with high promoter-driven intrinsic transcriptional noise
Source: BMC Evol Biol. 2014 Apr 4;14:74. doi: 10.1186/1471-2148-14-74 (PMC4031498; doi:10.1186/1471-2148-14-74)
Supplement: Additional file 1 — In Pdf format includes Supplementary Figures 1–6. [file 1471-2148-14-74-S1.pdf]

Zare et al. Sup. Figure 1

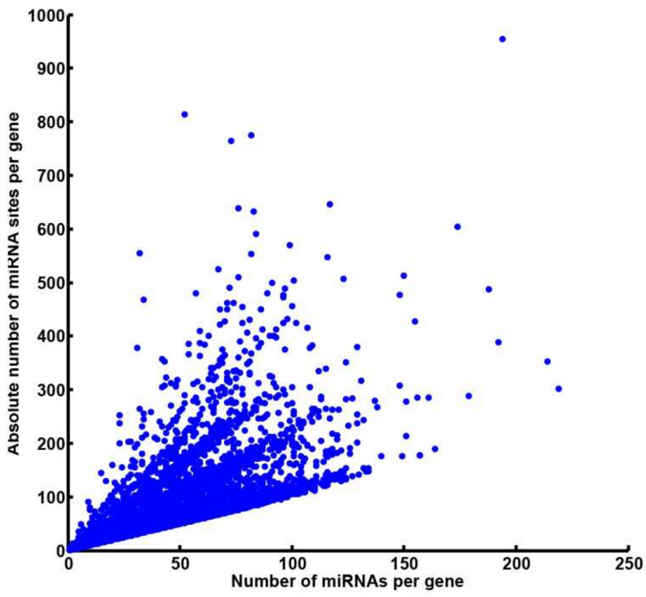

Figure S1. A scatter plot of number of miRNAs targeting 3'UTR (counting all occurrences of sites for the same miRNA as one) versus the absolute number of miRNA sites per gene. The 1st and 4th quartile groups of genes based on the absolute number of miRNA sites highly overlap with the 1st and 4th quartile groups studied in the paper based on mapping all occurrences of sites to one.

Zare et al. Sup. Figure 2

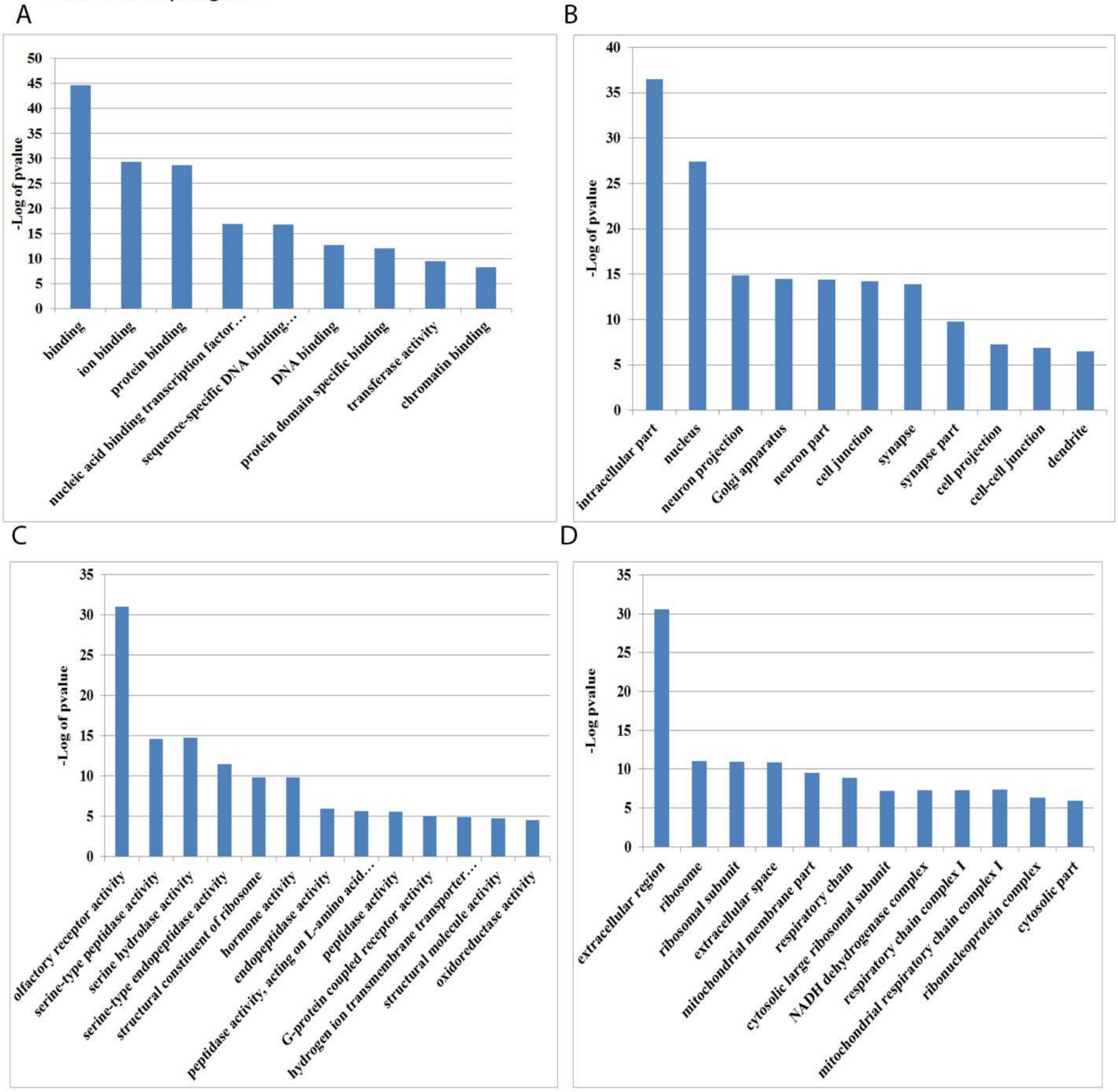

Figure S2. GO analysis of 1st and 4th quartile group. (A) Significantly enriched GO Molecular Function terms in 4th quartile. (B) Significantly enriched GO Cellular Component terms in 4th quartile. (C) Significantly enriched GO Molecular Function terms in 1st quartile. (D) Significantly enriched GO Cellular Component terms in 1st quartile.

Zare et al. Sup. Figure 3

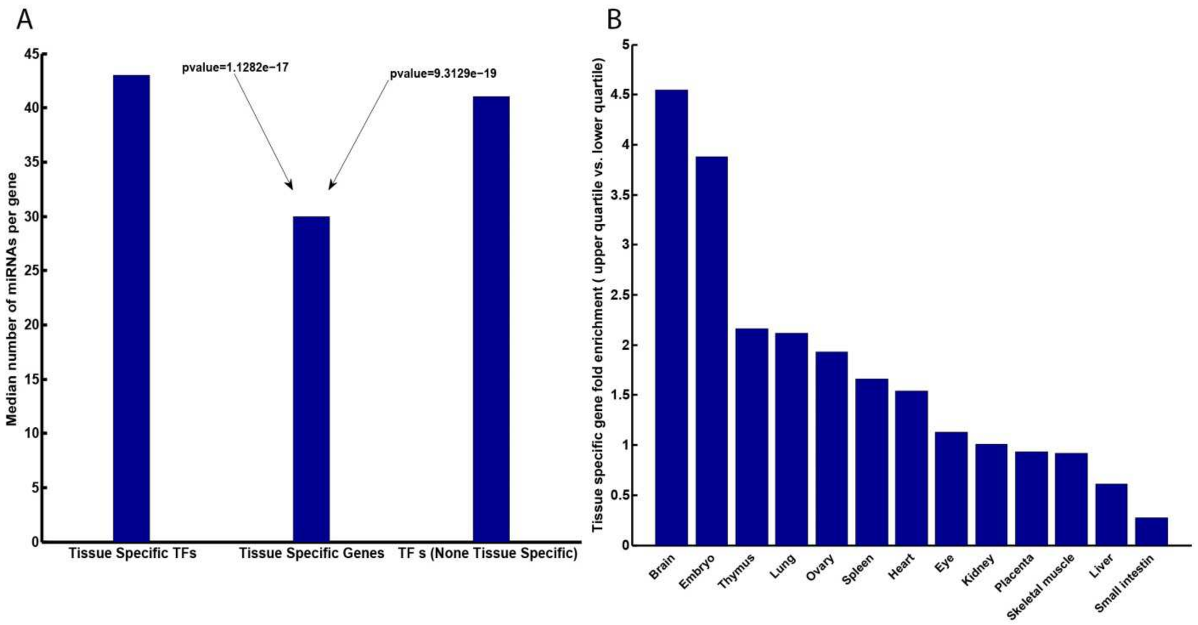

Figure S3. (A) Transcription factors on average are targeted by more miRNAs compared to tissue specific genes. (B) Enrichment comparison of tissue specific genes in 1st and 4th quartiles, Brain specific genes are more enriched in 4th quartile, i.e. they are targeted by high number of miRNAs.

Zare et al. Sup. Figure 4

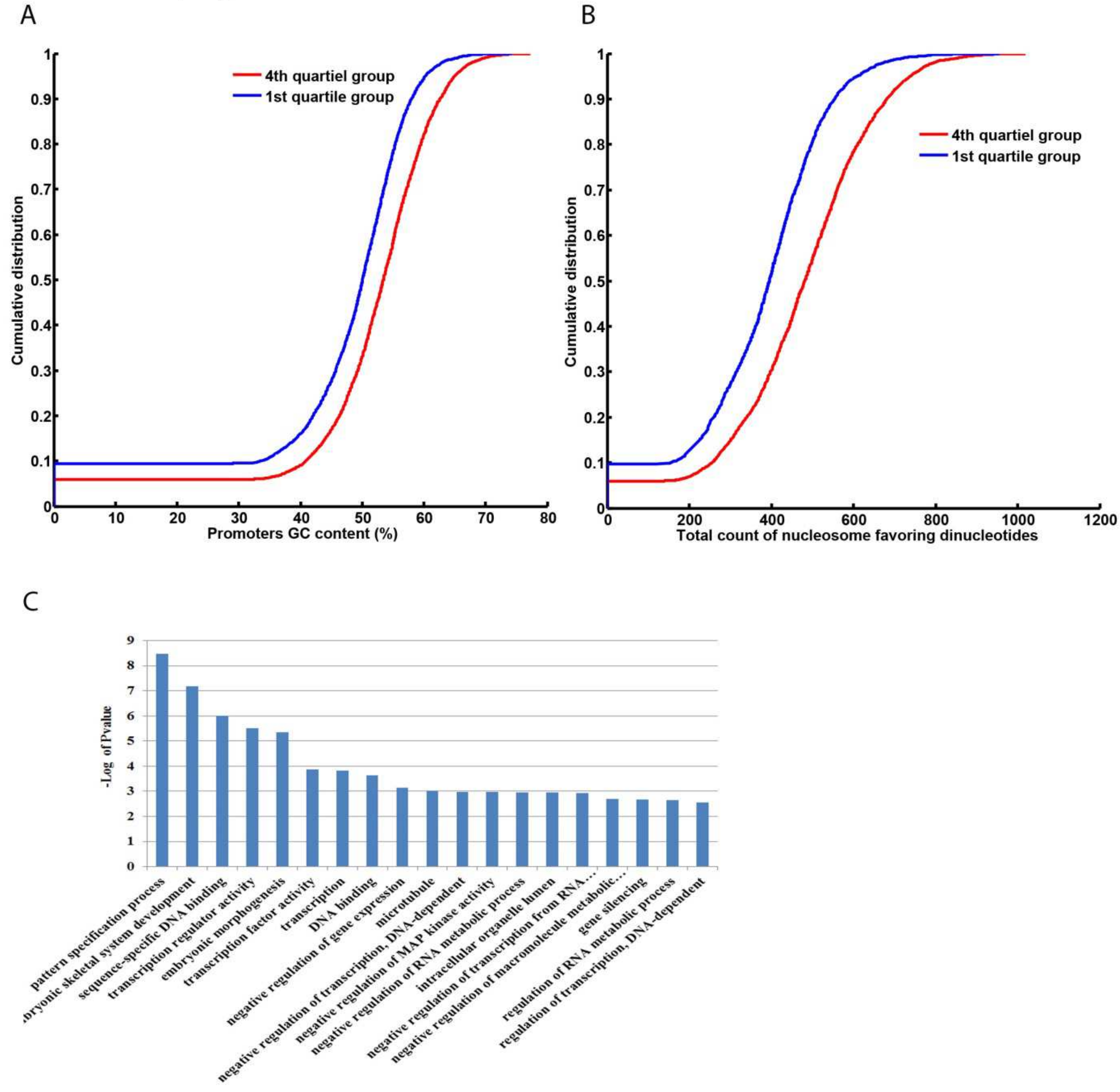

Figure S4. (A) The distribution of promoter GC content of genes in 1st (blue) and 4th (red) quartile group. (B) The distribution of number of nucleosome favoring di-nucleotides in promoters of genes in 1st (blue) and 4th (red) quartile groups. Data suggest that promoter sequence content of genes in 4th quartile favor nucleosome occupancy which can result in higher transcription noise. (C) GO analysis of genes co-localized with miRNAs, which indicate that most of them are involved in regulatory processes.

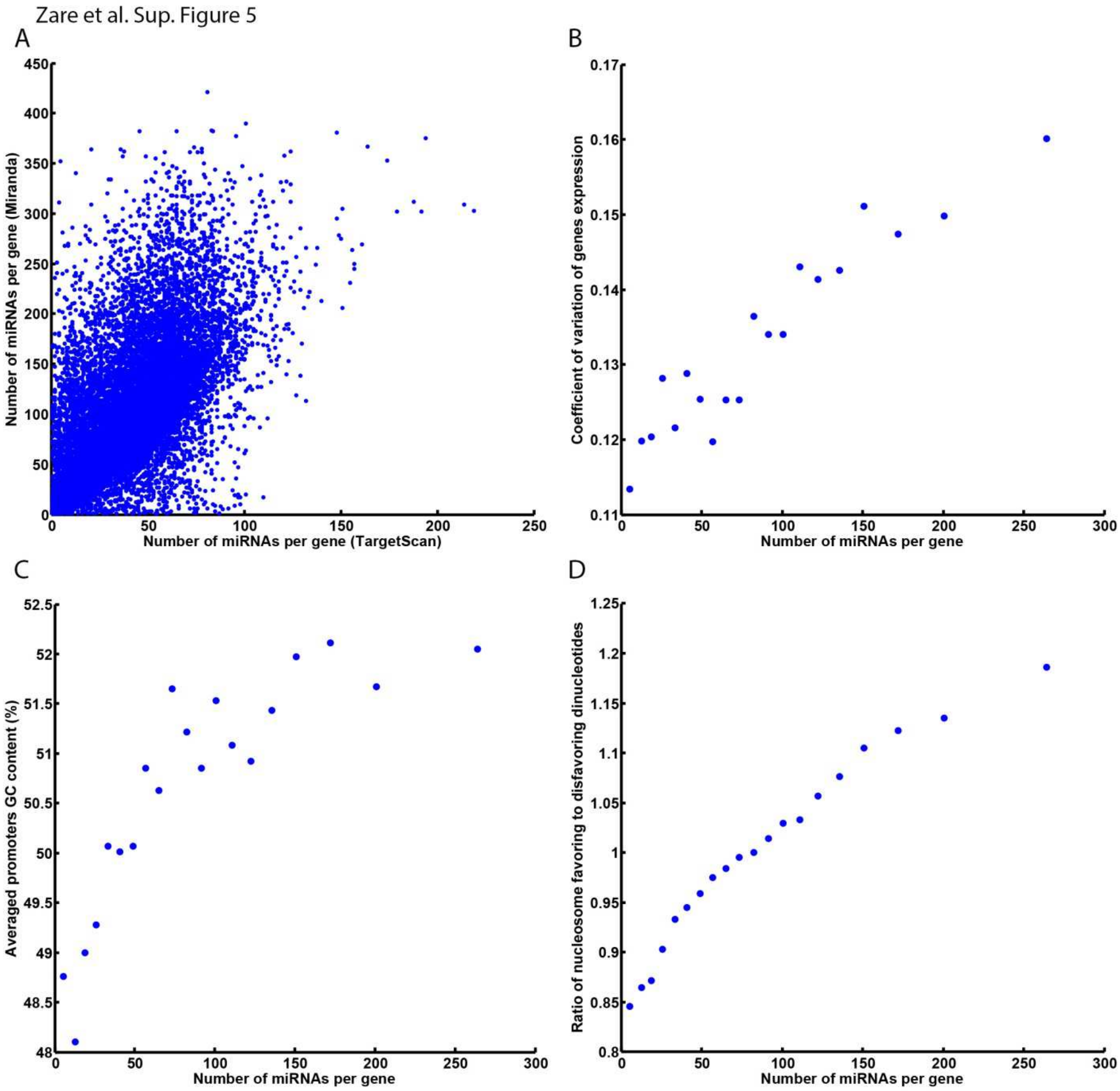

Figure S5. Similar analysis to that of Figure 3 using miRanda predicted sites instead of sites from TargetScan. (A) A scatter plot of the number of miRNA sites predicted using TargetScan and miRanada algorithms. (B) A scatter plot of the average gene expression variability versus the average number of distinct miRNA sites for vigintiles. Vigintiles were created by ranking genes based on the number of miRNA sites in their 3'UTR. (C) A scatter plot of the average promoter GC content versus the average number of distinct miRNA sites for vigintiles. (D) A scatter plot of the average ratio of nucleosome favoring to disfavoring di-nucleotides versus the average number of distinct miRNA sites for vigintiles.

Zare et al. Sup. Figure 6

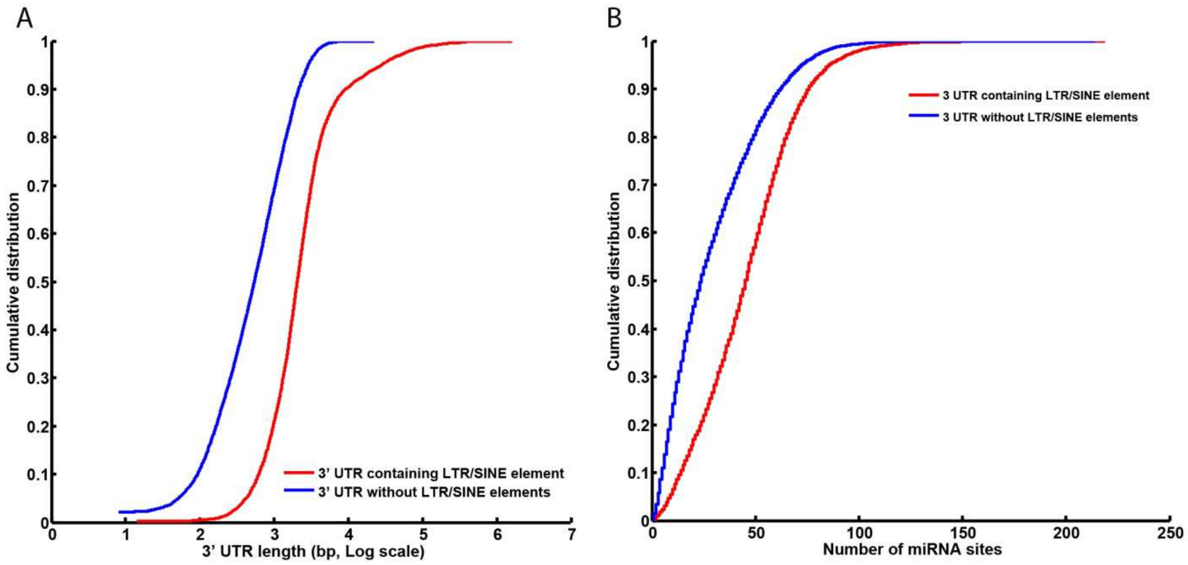

Figure S6. (A) 3' UTR containing repeat elements such as SINE/LTR are longer than those devoid of these elements. (B) 3' UTR containing LTR/SINE elements on average harbor more miRNA sites.
